# One-for-all gene inactivation *via* PAM-independent base editing in bacteria

**DOI:** 10.1016/j.jbc.2024.108113

**Published:** 2024-12-18

**Authors:** Xin Li, Ying Wei, Shu-Yan Wang, Shu-Guang Wang, Peng-Fei Xia

**Affiliations:** 1 School of Environmental Science and Engineering, Shandong University, Qingdao, China; 2 Sino-French Research Institute for Ecology and Environment, Shandong University, Qingdao, China; 3 Weihai Research Institute of Industrial Technology, Shandong University, Weihai, China

**Keywords:** CRISPR–Cas, base editing, SpRY, gene inactivation, bacteria

## Abstract

Base editing is preferable for bacterial gene inactivation without generating double-strand breaks, requiring homology recombination, or highly efficient DNA delivery capability. However, the potential of base editing is limited by the adjoined dependence on the editing window and protospacer adjacent motif. Herein, we report an unconstrained base-editing system to enable the inactivation of any genes of interest in bacteria. We employed a dCas9 derivative, dSpRY, and activation-induced cytidine deaminase to build a protospacer adjacent motif–independent base editor. Then, we programmed the base editor to exclude the START codon of a gene of interest instead of introducing premature STOP codons to obtain a universal approach for gene inactivation, namely XSTART, with an overall efficiency approaching 100%. By using XSTART, we successfully manipulated the amino acid metabolisms in *Escherichia coli*, generating glutamine, arginine, and aspartate auxotrophic strains. While we observed a high frequency of off-target events as a trade-off for increased efficiency, refining the regulatory system of XSTART to limit expression levels reduced off-target events by over 60% without sacrificing efficiency, aligning our results with previously reported levels. Finally, the effectiveness of XSTART was also demonstrated in probiotic *E*. *coli* Nissle 1917 and photoautotrophic cyanobacterium *Synechococcus elongatus*, illustrating its potential in reprogramming diverse bacteria.

Gene inactivation is essential for biological research and innovations. In bacteria, a predominant strategy is to disrupt the coding sequences (CDSs), which usually deploys a “dead-or-alive” selection. For the conventional knock-in method, bacteria have to integrate an antibiotic resistance gene into a certain genomic locus to survive the corresponding antibiotics, thus leading to the disruption of a CDS (1). This has been excelled by the revolutionary CRISPR–Cas system. The RNA-guided Cas nuclease finds a specific genomic locus and generates a double-strand break (DSB). When a repairing template (donor DNA) is supplied, the DSB will be repaired through homologous recombination (HR), resulting in living cells with designed and clean edits in the genome (2). Otherwise, the bacteria will die. Despite these advantages, the toxicity of Cas proteins, the low HR activity, and the requirement for efficient DNA delivery are making CRISPR–Cas-based gene inactivation challenging in bacteria (3, 4, 5).

Base editing deploys the CRISPR–Cas system and deamination for cytosine-to-thymine (C-to-T) or adenine-to-guanine (A-to-G) transitions in the genome at a single-nucleotide resolution (6, 7, 8). By using base editing, premature STOP codons can be introduced to genes of interest (GOIs) for gene inactivation (9). Notably, base editing employs a nuclease-deactivated Cas (dCas) protein with lower toxicity to bacteria, and it does not demand on HR for the desired editing, eventually relieving the reliance on highly efficient DNA delivery strategies to enable abundant transformants surviving from a “dead-or-alive” selection (3, 10). These advantages empower base editing as a preferable tool for gene editing, and its capacities have been demonstrated in various bacteria, including *Escherichia coli* (11), cyanobacteria (12, 13), acetogens (10) and marine bacteria (14).

Two intrinsic features, however, constrain the potential of base editing. First, as a CRISPR–Cas-based system, it relies on a specific protospacer adjacent motif (PAM). Second, it has a specific hot-spot editing window, within which highly efficient base editing can be achieved. For instance, a base editor employing dCas9 from *Streptococcus pyogenes* and the activation-induced cytidine deaminase (AID) from *Petromyzon marinus* recognizes an NGG PAM, and the hot-spot editing window lies between positions −16 and −19 in a spacer (the nucleotide next to the PAM as position −1) (15, 16). In addition, a premature STOP codon can only be introduced based on four codons (15). Taking together, to inactivate a GOI, we must find a specific codon (*e*.*g*., CAG coding for Gln) within a certain editing window (positions −16 to −19) of a spacer adjacent to a particular PAM (*e*.*g*., NGG), where the spacer is preferably located in the first half of the CDS to avoid unexpected truncations. Relieving these constraints would substantially enhance and expand the utility of base editing.

Here, we designed and established a one-for-all gene inactivation strategy for bacteria with unconstrained base editing. First, we employed a dCas9 derivative, dSpRY (17, 18), as the effector to build a cytosine base editor with AID, releasing the dependence on PAM. Then, we programmed the base editor to exclude the START codon (*e*.*g*., ATG and GTG) of a GOI instead of introducing STOP codons, namely XSTART. By using XSTART, we successfully manipulated the amino acid metabolisms in *E*. *coli*, generating glutamine, arginine, and aspartate auxotrophic strains. Furthermore, we attempted to limit the XSTART expression to reduce off-target events. The capability of XSTART was also demonstrated in probiotic *E*. *coli* Nissle 1917 (EcN) and photoautotrophic cyanobacteria.

## Results

### Design of a PAM-independent base editor for bacteria

**Figure 1:**
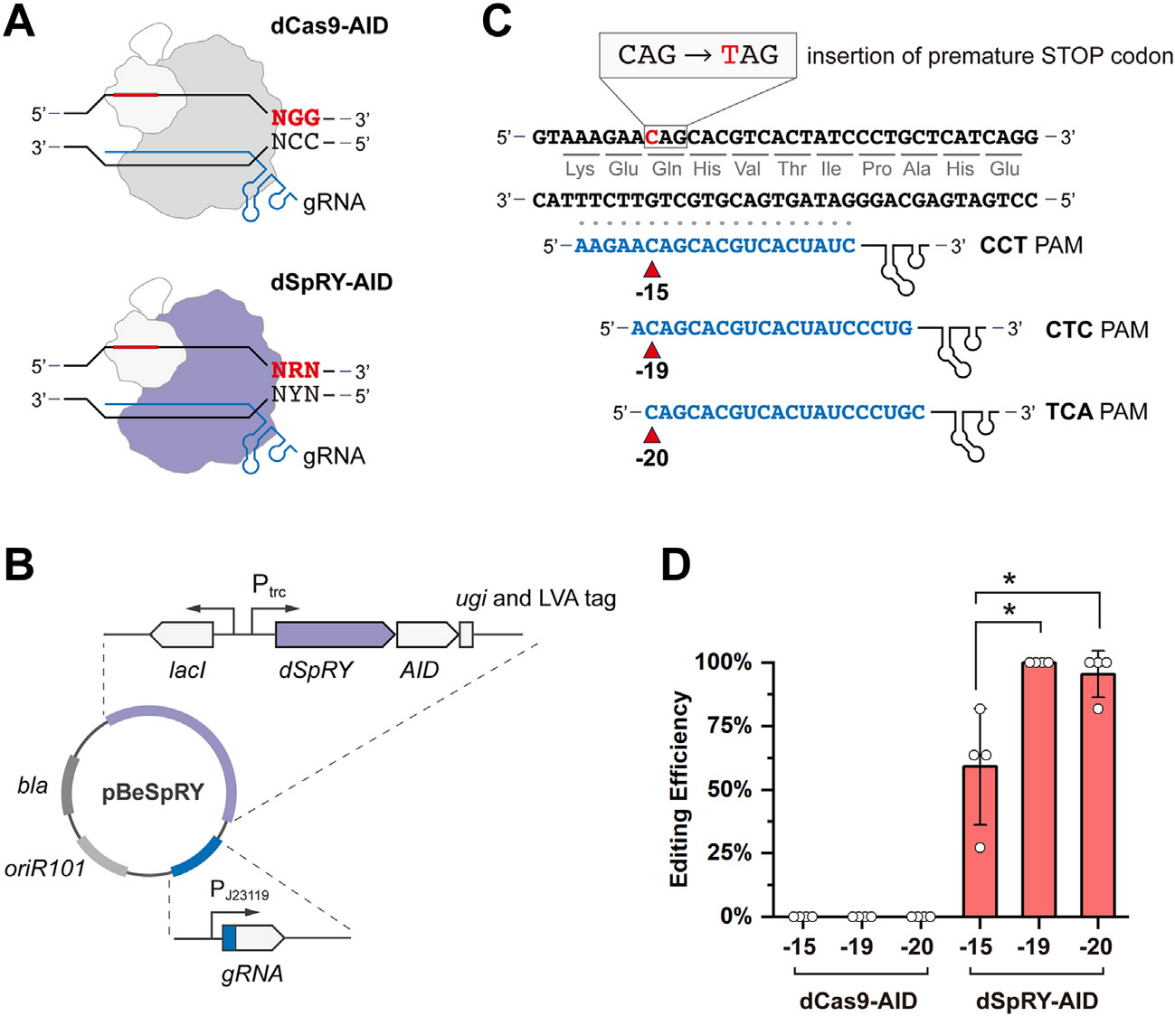
**Unconstrained base editing with dSpRY**. *A*, schematic illustration of dCas9–AID and dSpRY–AID mediated base editing, where dCas9–AID recognizes an NGG PAM and dSpRY–AID recognizes an NRN or NYN PAM. R stands for A or G, and Y stands for T or C. *B*, design of the dSpRY–AID plasmid pBeSpRY. It contains the dSpRY–AID module carrying *dSpRY*, *AID*, *ugi*, and LVA tag driven by a *lacI-*P_trc_-inducible system. The gRNA cassette is under the control of the constitutive promoter P_J23119_. *C*, design of the three spacers targeting *glnA* based on dSpRY–AID editing module, where the targeted C was relocated in different editing windows with unconstrained PAM. The *red arrows* indicate the designed editing loci. *D*, base editing efficiencies of dCas9–AID and dSpRY–AID systems. The results represent the means of four biologically independent replicates, and the error bars indicate the SD. The differences were statistically evaluated by *t* test (∗*p* < 0.05, unpaired and two-tailed). AID, activation-induced cytidine deaminase; gRNA, guide RNA; LVA, Leu-Val-Ala; PAM, protospacer adjacent motif.

SpRY is an engineered Cas9 derivative, finding a protospacer without the requirement of PAM, whereas a preference for NRN (R stands for A or G) over NYN (Y stands for T or C) PAM was reported (Fig. 1*A*) (18). We first modularly assembled the dSpRY (D10A and H840A) and AID from *P*. *marinus* as the editing module with uracil DNA glycosylase inhibitor and Leu-Val-Ala degradation tag to enhance the performance (11). Then, the guide RNA (gRNA) was implemented. Finally, we chose a temperature-sensitive replication origin for selecting, maintaining, and rescuing the working plasmids (Fig. 1*B*). Presumably, the resulting dSpRY–AID system would release its requirement of PAM, and with such a system, we can relocate the target loci to the hot-spot editing window of the base editor for improved editing efficiency.

To demonstrate the system, we chose *glnA*, encoding the glutamine synthetase, as a target, and attempted to insert a premature STOP codon in Q30 (Fig. 1*C*). We designed three gRNAs (Table S1) with the target locus located in positions −15, −19, and −20, respectively, with corresponding PAMs CCT, CTC, and TCA (Fig. 1*C*). Meanwhile, a dCas9–AID system was generated for comparison with the same gRNAs (Fig. S1). As expected, the dCas9–AID system showed no editing efficacy with these non-NGG PAMs (Fig. 1*C*). To the contrary, the dSpRY–AID system showed promising activity, even when the target nucleotide was located in position −15. We observed that when the loci of the target moved to positions −19 and −20, the editing efficiencies significantly increased reaching 100% and 95.45 ± 9.09%, indicating the capacity of dSpRY–AID for improving editing performance (Fig. 1*D*). To be noticed, we also found mixed signals in the resulting colonies at the first round of selection, which is a common issue for base editing (10, 14), while strains with pure edits could be obtained *via* one more round of segregation (Fig. S2).

### One-for-all gene inactivation by excluding the START codon (XSTART)

**Figure 2:**
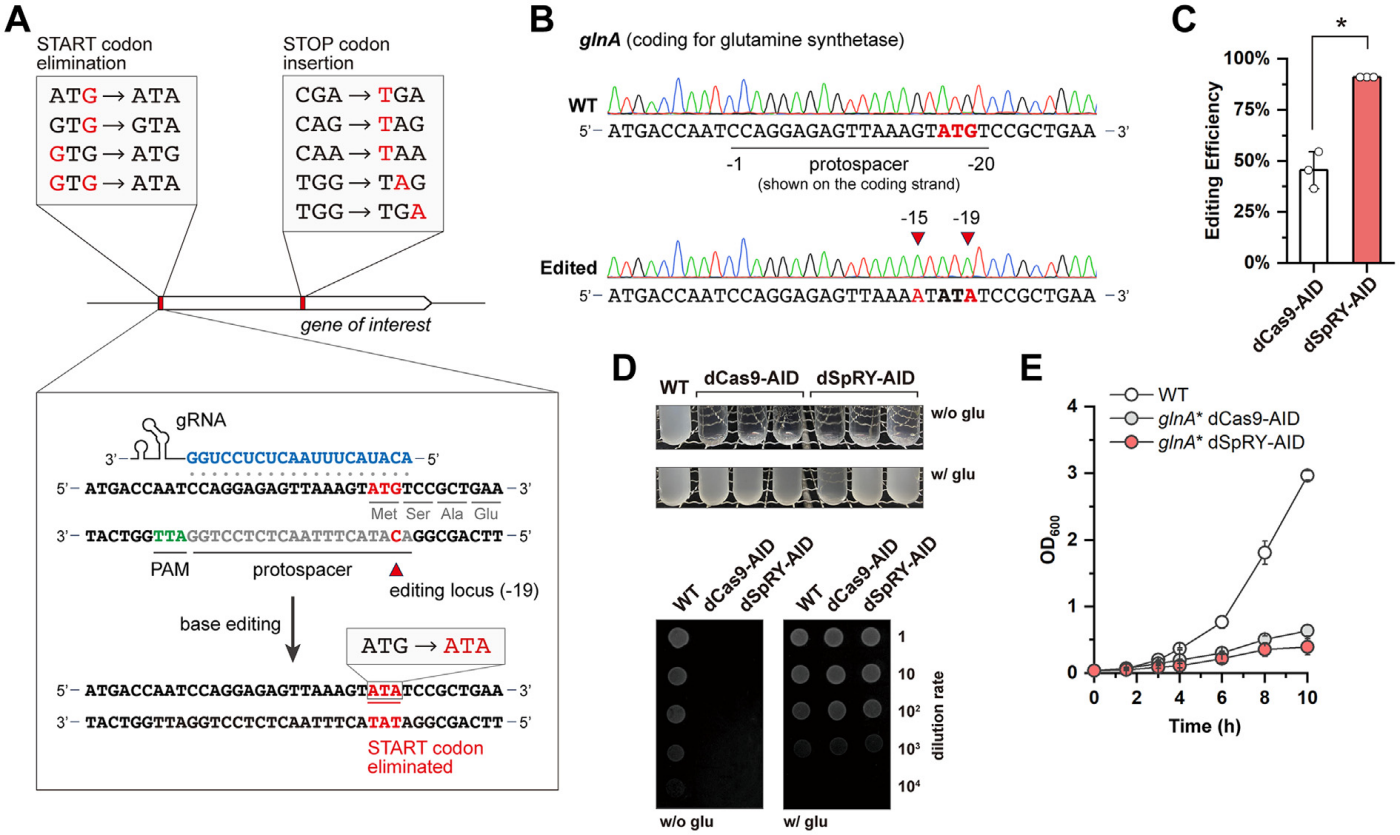
**Design principle and demonstration of XSTART**. *A*, principles of XSTART. The designed strategy, in theory, can inactivate any GOIs by designing a gRNA targeting the noncoding strand with CAT or CAC (the reverse complement of the START codon ATG or GTG) located in the hot-spot editing window without considering any specific PAM. *B*, sequencing results of *glnA* in the wildtype and the edited strain with XSTART. The edited loci are indicated by *red arrows* and highlighted in *red*. *C*, editing efficiencies of XSTART with dCas9–AID and dSpRY–AID as effectors. The results are shown as the means of three biologically independent experiments. The error bars indicate the SDs, and the differences were statistically evaluated by *t* test (∗*p* < 0.05, unpaired and twotailed). *D*, phenotypical evaluation of the *glnA*-inactivated strain. For each edited strain, we randomly selected three independent colonies for phenotypical evaluation in liquid medium, while we picked one colony for the spot assay. *E*, growth profiles of the *glnA*-inactivated strains. Experiments were performed with three independent replicates, and the error bars indicate the SDs. All strains were evaluated after plasmid curing. AID, activation-induced cytidine deaminase; GOI, gene of interest; PAM, protospacer adjacent motif.

Only four specific codons can be converted to the three STOP codons, but the START codons, which intrinsically contain the nucleotide G, are universal and can be eliminated with merely one C-to-T transition on the noncoding strand (Fig. 2*A*). The resulting DNA sequence has, if any, limited influences as translation will not initiate anymore. Therefore, we designed XSTART for gene inactivation by excluding the START codons with dSpRY–AID. Theoretically, XSTART can edit any GOI by designing a gRNA targeting the noncoding strand with CAT or CAC (the reverse complement of the START codon ATG or GTG) located in the hot-spot editing window without considering any specific PAM. As a proof of concept, we designed gRNA04 (Table S1) with an ATT PAM, targeting the noncoding strand of *glnA*, where the nucleotide C in CAT was located in position −19 for maximal editing efficiency (Fig. 2*A*). A successful editing would lead to the elimination of the START codon, thus inactivating *glnA*.

As a result, the START codon was successfully excluded, changing ATG to ATA (Fig. 2*B*), and we observed an identical editing efficiency of 90.91% across all three individual rounds of experiments (Fig. 2*C*). We found bystander editing at position −15 of the spacer where the G in the 5′-untranslated region was converted to A (a C-to-T editing on the noncoding strand), but it would not influence the efficacy of our system (Fig. 2*A*). Interestingly, we found that XSTART could also work with dCas9–AID with a non-NGG PAM for this specific target, which may result from the TGG motif next to the designed ATT PAM (Fig. 2*A*). This agreed with a previous report using dCas9-mediated base editing to eliminate START codons in rabbit models, but an NGG or NGN PAM is compulsory (19). We compared the editing efficiencies of these two systems in *E*. *coli* and found that dSpRY–AID had a significantly higher efficiency compared with that of dCas9–AID (45.45 ± 9.09%) (Fig. 2*C*). This, as designed, can be explained by the released dependence on PAM and the relocation of the target nucleotide C in the preferred editing window. Next, we examined the phenotypical changes of the edited strains both in liquid medium and agar plates. As expected, the edited strains cannot grow in minimal medium without the supplement of glutamine (Fig. 2, *D* and *E*), indicating that XSTART could be a promising strategy for gene inactivation.

### Reprogramming amino acid metabolism in *E*. *coli* with multiplex XSTART

**Figure 3:**
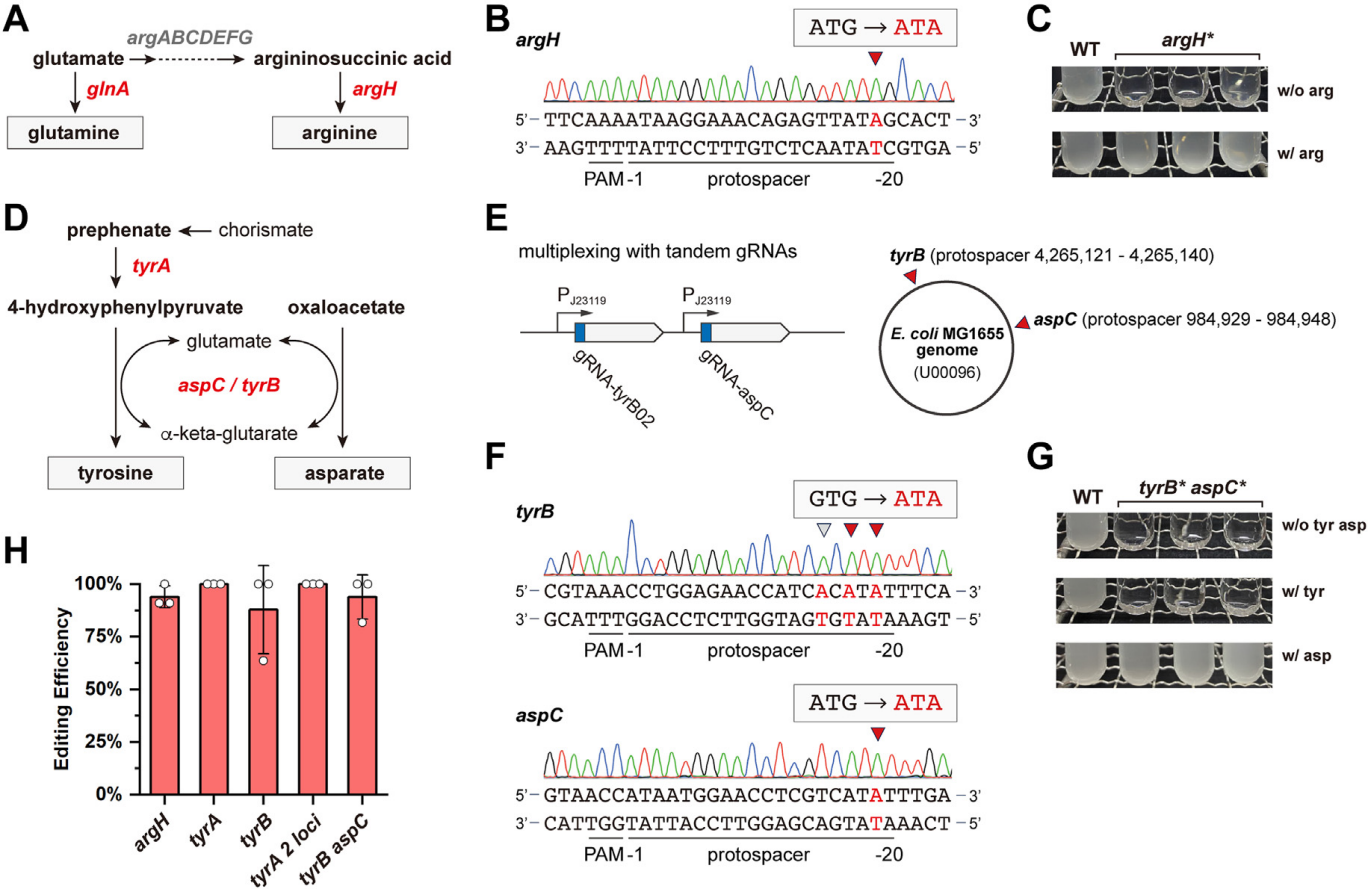
**Perturbation of the amino acid metabolisms in *Escherichia coli* with multiplex XSTART**. *A*, schematic illustration of the glutamine and arginine metabolisms in *E*. *coli*. The *argH* gene encoding for the argininosuccinate lyase and *glnA* encoding for the glutamine synthetase are highlighted. *B*, sequencing results of *argH* edited with XSTART. *C*, phenotypical evaluation of *argH*-inactivated strain. For phenotypical evaluation, we randomly selected three independent colonies from each edited strain. *D*, schematic illustration of the tyrosine and aspartate metabolisms in *E*. *coli*. The *tyrA* gene encoding for the fused chorismate mutase T or prephenate dehydrogenase, the *aspC* gene encoding for the aspartate aminotransferase, and the *tyrB* gene encoding for tyrosine aminotransferase are highlighted. *E*, design of the tandem gRNAs (gRNA07 and gRNA09) targeting *tyrB* and *asp*C simultaneously. The tandem gRNA cassette is under the control of the constitutive promoter P_J23119_. The loci of protospacers in *tyrB* and *aspC* in the *E*. *coli* MG1655 genome are indicated by *red arrows*. *F*, sequencing results of *tyrB* and *aspC* edited by the multiplex XSTART. *G*, phenotypical evaluation of the *tyrB* and *aspC* double inactivated strain. For phenotypical evaluation, we randomly selected three independent colonies from each edited strain, and all tested strains were plasmid cured. *H*, editing efficiencies of XSTART in *E*. *coli*. The means of the editing efficiencies from three independent replicates are shown. The edited loci are indicated by *red arrows* and highlighted in *red*. Three independent isolates of the edited strain were randomly chosen for phenotypical evaluation. gRNA, guide RNA.

Next, we attempted to reprogram the amino acid metabolisms of *E*. *coli* with XSTART. While we already demonstrated that XSTART can inactivate *glnA* and generate a glutamine auxotrophic strain, we selected the arginine metabolism as a second target. The inactivation of *argH*, coding for the argininosuccinate lyase, would result in an arginine auxotrophic strain (Fig. 3*A*). With gRNA05 targeting the START codon of *argH*, we obtained efficient conversion of ATG to ATA, excluding the START codon (Fig. 3*B*), and the resulting strain can only grow in minimal medium with the supplement of arginine (Figs. 3*C* and S3). Then, we tried to generate a tyrosine auxotrophic strain by inactivating *tyrA*, coding for the fused chorismate mutase T or prephenate dehydrogenase, and *tyrB*, coding for the tyrosine aminotransferase, respectively (Fig. 3*D*). Though we found successful elimination of the START codon of *tyrA*, the edited strain could still grow without tyrosine (Fig. S4), and similar success in genome editing but failure in phenotypical verification was observed for *tyrB* (Fig. S5).

We closely checked the CDS of *tyrA* and found another ATG located downstream of the original START codon (Fig. S6). Therefore, we hypothesized that the resulting strain might contain an N terminus-truncated *tyrA* rather than an inactivated one. To check this assumption, we generated a multiplex XSTART system *via* assembly of tandem gRNAs (gRNA06 and gRNA08) targeting the two ATGs at once. As expected, the two loci were both edited, and the ATGs were removed (Fig. S6). But still, the resulting strain can grow without tyrosine, indicating alternative pathways supplying tyrosine for cell growth. As previously reported, *tyrB* and *aspC* (coding for aspartate aminotransferase) share similar catalytical activities, the function of which can be complemented by each other (20). So, we deployed the multiplex XSTART for the inactivation of both genes with gRNA07 and gRNA09 (Fig. 3*E*). We successfully obtained the strain with both genes edited (Fig. 3*F*). Surprisingly, we found that the resulting strain with the deficient *tyrB* and *aspC* cannot grow in minimal medium with or without tyrosine, but it could survive the minimal medium with the supplement of aspartate, giving an aspartate auxotrophic strain (Figs. 3*G* and S3). This is in line with a previous study discussing amino acid metabolisms in *E*. *coli* (21), but the mechanism remains unclear. It was worth noticing that the editing efficiencies of XSTART were overall above 87.88% for all the aforementioned experiments (Fig. 3*H*), indicating the grand potential of this universal strategy. The unexpected phenotypes of the edited strains may be due to the complex metabolisms of amino acids (21, 22).

### Trade-offs between universality and precision

**Figure 4:**
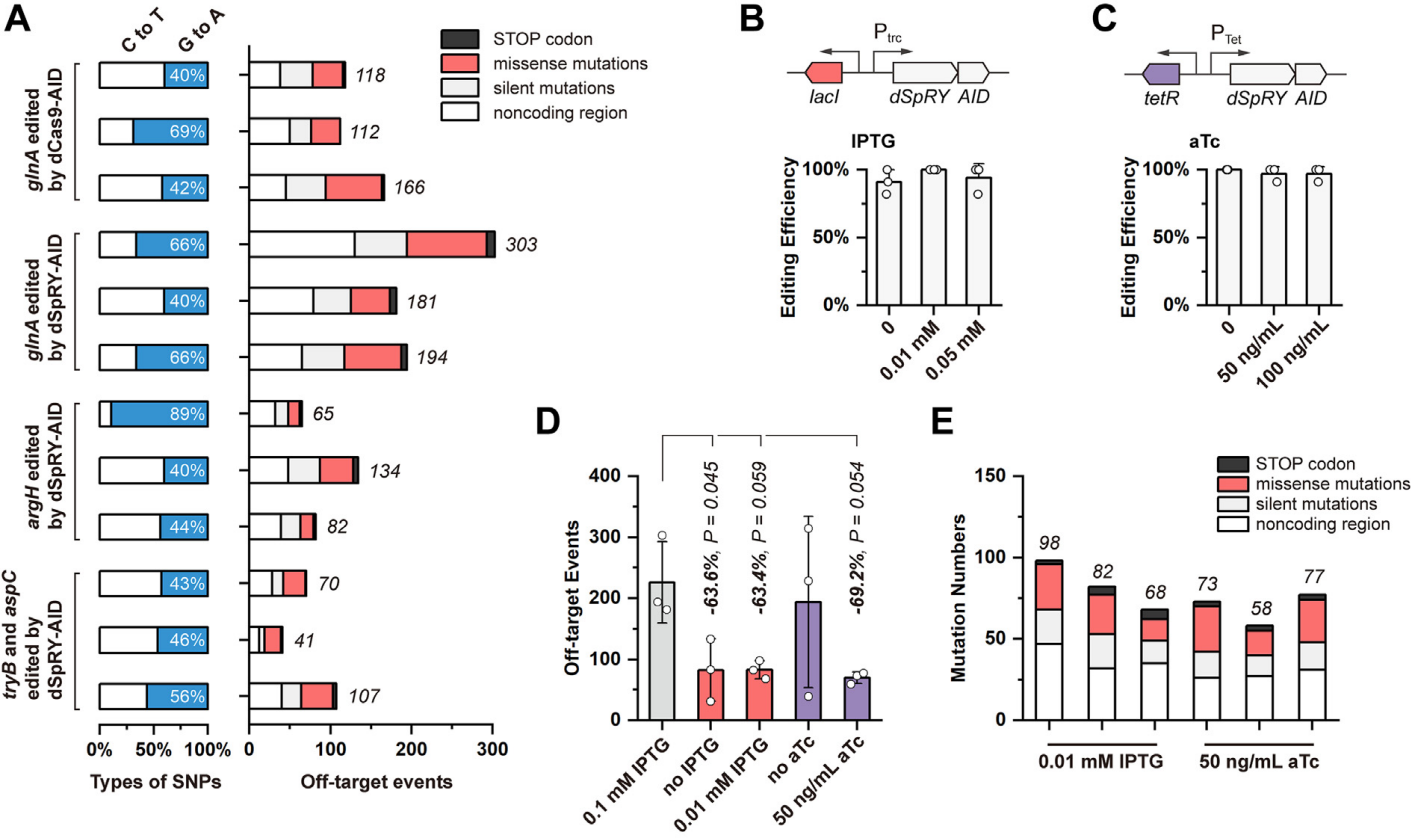
**Alleviating the off-target events by limiting XSTART expression**. *A*, the distribution of different types of SNPs and the consequential mutations are shown, including missense (*red*), nonsense (premature STOP codon, *black*), silent mutations (*light gray*), and the mutations that did not lie in the CDS (*white*). *B* and *C*, illustrates the design of two different inducible systems with *lacI*-P_trc_ and *tetR*-P_tet_, respectively, and the corresponding editing efficiencies at different dosages of inducers. *D*, numbers of off-target events of the two systems at two different concentrations of IPTG and aTc, respectively. The results were compared with that of the *lacI*-P_trc_ system with 0.1 mM IPTG. The differences were statistically evaluated by *t* test (unpaired and twotailed). *E*, the distribution of different types of SNPs and the mutations are shown following the same format of *A*. All off-target evaluations were performed on three independent isolates for each strain *via* whole-genome sequencing. aTc, anhydrotetracycline; CDS, coding sequence.

Unlike conventional CRISPR–Cas-based genome editing, base editing leads to unwanted off-target events. Therefore, we performed whole-genome sequencing to analyze the off-target events caused by XSTART. Four edited strains have been selected, each with three biological independent colonies, including *glnA* edited by dCas9–AID, by dSpRY–AID, *argH* edited by dSpRY–AID, and *tryB* and *aspC* edited by dSpRY–AID (Fig. 4*A*). According to the whole-genome sequencing data, we found unexpected high frequency of off-target events comparing to previous reports using AID from *P*. *marinus* (11, 12, 14). Especially, we observed only less than 10 off-target events when inserting premature STOP codons in *S*. *elongatus* (12) and *Roseovarius nubinhibens* (14). The off-target events of XSTART ranged from 41 to 303 with an average of around 130 counts, and all were C-to-T or G-to-A transitions (Fig. 4*A* and Dataset 1). No statistical differences were noted among the four different editings (Dataset-1), while the abnormally high off-target events (303) only occurred in one colony with edited *glnA* (Fig. 4*A*). To be noticed, the numbers of the off-target events between dSpRY–AID and dCas9–AID were also not statistically different (*p* = 0.12), whereas that of dSpRY–AID was slightly higher, indicating that the high frequency of off-target events resulted from the targeting of START codons rather than the released PAM dependency.

We hypothesized that the increased off-target editing by XSTART might be an inevitable trade-off for its high efficiency. To balance efficiency and precision, we attempted to limit the expression level of XSTART with lower levels of inducers (0, 0.01, and 0.05 mM IPTG), achieving efficiencies over 90% (Fig. 4*C*), comparable to that of 0.1 mM IPTG used previously (Fig. 2*C*). We further built a *tetR*-P_tet_-inducible system for tighter regulation, using anhydrotetracycline (aTc) at 0, 50, and 100 ng/ml, which yielded near 100% editing efficiency (targeting *glnA* with gRNA04) (Fig. 4*C*). Next, we analyzed off-target effects in four *glnA*-edited strains induced with 0 and 0.01 mM IPTG and 0 and 50 ng/ml aTc. Three colonies of each edited strain were randomly selected for evaluation. While efficiency remained unaffected, off-target events decreased by 63.6%, 63.4%, and 69.2% with 0, 0.01 mM IPTG and 50 ng/ml aTc, respectively (Fig. 4*D*). However, off-target counts in the strain edited without aTc fluctuated ranging from 38 to 313 events (Fig. 4*D*), where the abnormally fluctuating off-target events might result from unintended leaky expression of the *tetR*-P_tet_-inducible system without aTc.

Detailed analysis of mutations generated with 0.01 mM IPTG and 50 ng/ml aTc, which led to the lowest and most stable results, revealed totals ranging from 58 to 98, slightly higher but comparable to conventional base editing (Table S2). Among these, 68.7 ± 3.1% and 62.9 ± 4.7% were noncoding or silent mutations (Fig. 4*E*). Fewer than six early STOP codons and 13 to 28 missense mutations were identified in a single tested strain (Dataset 2). The results highlighted the need for comprehensive evaluation of edited strains when high precision is essential, although no impacts on our intended editing were observed. Closer examination indicated that most unintended mutations stemmed from random deamination (Datasets 1 and 2), with two shared off-target editing in *apaG* and *uidB* likely because of unintended CRISPR targeting. In *apaG*, we found an identical sequence to the spacer of gRNA04, whereas *uidB* has a continuous 11 bp sequence same to the spacer. This agrees with previous reports (23, 24) and may partially explain the trade-off between efficiency and precision. Taking together, limiting expression levels effectively reduced off-target events by over 60% without compromising efficiency, and the *lacI-P*_*trc*_-inducible system would be a preferable system with promising and stable performance, unconstraining the capability of XSTART as a universal gene inactivation strategy.

### Generalizability of XSTART

**Figure 5:**
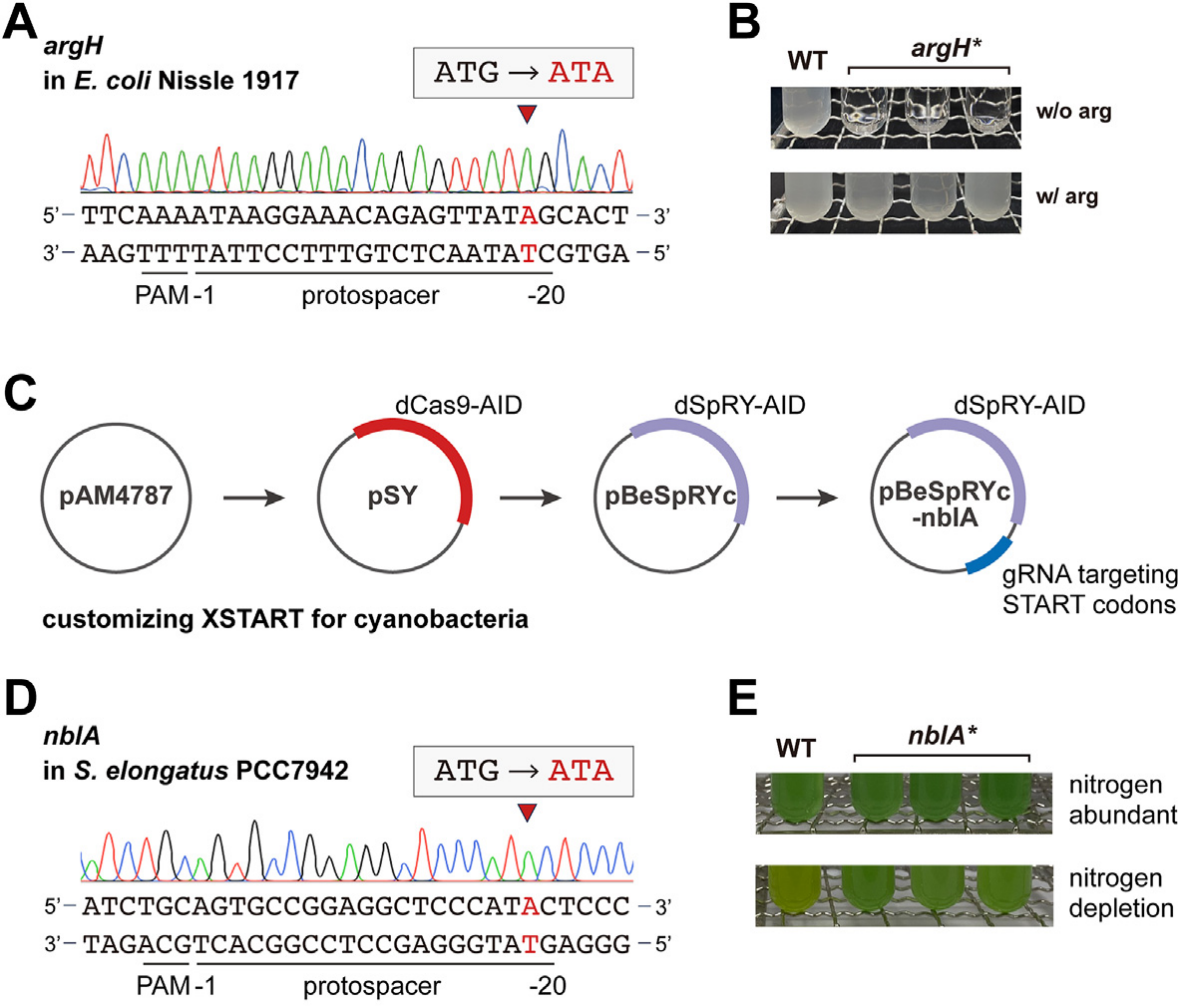
**Base editing in EcN and cyanobacterium *Synechococcus elongatus* PCC7942 with XSTART**. *A*, sequencing results of *argH* in EcN edited by XSTART with pBeSpRY-argH. *B*, phenotypical evaluation of the *argH*-inactivated EcN. *C*, customization of XSTART for cyanobacteria *via* replacing the dCas9–AID module on pSY plasmid with the dSpRY–AID module. *D*, sequencing results of *nblA* in *S*. *elongatus* PCC7942. *E*, phenotypical evaluation of the *nblA*-inactivated *S*. *elongatus* PCC7942 in nitrogen-rich and depletion conditions. The edited loci are indicated by *red arrows* and highlighted in *red*. Three independent isolates of the edited strain were randomly chosen for phenotypical evaluation, and all tested strains were plasmid cured. AID, activation-induced cytidine deaminas; EcN, *E*. *coli* Nissle 1917.

Moreover, we selected the probiotic EcN and cyanobacterium *S*. *elongatus* PCC7942 to demonstrate the universality of XSTART. As EcN is a clinical isolate of *E*. *coli* (25), we deployed the same system as for the model strain and targeted the *argH* gene to generate an auxotrophic EcN. The resulting strain would be useful as a probiotic chassis to harbor functional plasmids with auxotrophic markers for selection, avoiding the utilization of antibiotic resistance genes (26, 27). By using XSTART, we managed to inactivate *argH* in EcN (Fig. 5*A*), and the edited strain can only grow in a minimal medium with the supplement of arginine (Fig. 5*B*).

To allow XSTART work for *S*. *elongatus*, we generated a working plasmid *via* updating our previously established pSY serial plasmids with dSpRY–AID substituting dCas9–AID on the pAM4787 backbone (Fig. 5*C*) (12, 28). Then, we designed XSTART by targeting the START codon of *nblA*, coding for the phycobilisome hydrolyzing enzyme, with gRNA10 (Fig. 5*D*). The *nblA-*inactivated *S*. *elongatus* will no longer exhibit the bleaching phenotype and remain green under nitrogen-limited condition, whereas the wildtype strain tends to turn to yellow without nitrogen (29). With the customized XSTART for *S*. *elongatus*, we successfully inactivated *nblA* (Fig. 5*D*), and the variation of culture color was observed with the gene-edited strain under nitrogen-limited condition (Fig. 5*E*). These results demonstrated the generalizability of XSTART in different strains *via* modular assembly of the essential modules and programming gRNAs with a straightforward design principle.

## Discussion

Here, we report a one-for-all gene inactivation strategy, XSTART, for bacteria leveraging dSpRY-driven base editing. SpRY, the engineered Cas9 derivative, exhibited unusual advantages for developing base editors because of the independence of PAMs, thus significantly expanding the utility of base editing (17, 18, 30). In return, a base-editing system using dSpRY does not cut DNAs, avoiding the cleavage of DNA coding for gRNAs. This is because SpRY can hardly distinguish the target DNA sequence from the gRNA sequence on plasmid, and we did observe mutations in the gRNA region of our working plasmids (Fig. S7). After releasing the PAM dependence, we designed XSTART to mutate the START codon rather than to insert STOP codons. A START codon intrinsically exists in CDS coding for the first amino acid of a protein, such as ATG or GTG. As long as one of the Gs is converted, a START codon can be eliminated. To the contrary, one must find the four specific codons for the insertion of premature STOP codons, which is sometimes difficult or impossible. Therefore, the combination of PAM-independent base editing and the principle of START codon exclusion finds a unique but wide niche for gene inactivation in bacteria.

We noticed that recent studies also recognized similar potentials of unconstrained base editing in bacteria. Two reports described a strategy by using cytosine base editor to insert STOP codons and using adenine base editor to manipulate START codons for efficient gene editing in *Bacillus subtilis* and *Pseudomonas putida*, respectively (31, 32). An earlier study in two rabbit models utilized dCas9-based base editor to mutate START codons, but an NGG or NGN PAM is compulsory, limiting its utility (19). Differently, our XSTART, with a single base-editing system, can be a one-for-all strategy that is capable of inactivating any GOIs by simply designing a gRNA targeting the universal START codons from the noncoding strand, neither switching between different types of base editors, calculating the PAM and editing window nor digging for the candidate codons to introduce premature STOP codons.

One essential feature is that base editing bypasses the strong “dead-or-alive” selection from DSBs, making it preferable for bacteria that are sensitive to Cas nuclease and lack efficient HR. However, without a DSB, off-target events become inevitable, as an off-target binding of base editor will not kill the bacteria anymore. This is actually true and has been carefully analyzed as reported previously in, to name a few, *S*. *elongatus* (12), *Streptomycetes* (33), *R*. *nubinhibens* (14), *Clostridium autoethanogenum* (34) and *P*. *putida* (35). According to the literature and our previous work, base editors with AID showed countable off-target events and most of them resulting from the deamination (11, 12, 14), where C-terminal APOBEC1 (apolipoprotein B mRNA-editing enzyme)-based system showed higher but acceptable frequency of off-target events (23, 24, 33). Though XSTART showed great capability of gene inactivation, a price of higher frequency of off-target events has been paid. We observed larger number of off-target editing using XSTART comparing to early STOP codon insertion using similar base editors. While we successfully reduced the off-target events by over 60% without compromising efficiency, a slightly elevated level of off-target effects remains, which might be an inevitable trade-off for higher efficiency and broader applicability. To tackle this issue, innovations with fundamental advancements are on demand.

## Experimental procedures

### Strains and media

All strains used in this study are listed in Table S3. *E*. *coli* DH5α (Takara Bio Tech) was used for molecular cloning to construct plasmids. *E*. *coli* MG1655 (CGSC#6300) strain, EcN strain, and cyanobacteria *S*. *elongatus* PCC7942 (American Type Culture Collection 33912) strain were used to test the feasibility of base-editing system. EcN was a generous gift from Dr Chun Loong Ho. Both *E*. *coli* strains were cultivated in LB medium (5 g/l yeast extract, 10 g/l tryptone, 10 g/l NaCl; solid medium with 1.5% agar) supplemented with ampicillin (150 μg/ml) or spectinomycin (60 μg/ml) when appropriate. All *E*. *coli* strains were cultivated at 37 °C, whereas MG1655 carrying plasmids with the temperature-sensitive origin of replication were grown at 30 °C. Cyanobacteria were cultivated in standard or nitrate-depleted BG-11 medium (solid medium with 1.5% agarose) at 30 °C with continuous illumination (30–40 μmol protons m^-2^⋅s^-1^) (12). When necessary, spectinomycin (2 μg/ml) and streptomycin (2 μg/ml) were added to the medium. IPTG (0.1 mM) was added to the medium to induce base editing.

### Plasmid construction

To build the editing plasmid pBeSpRY for *E*. *coli*, we first chose the pKD46 plasmid with temperature-sensitive origin of replication, thus allowing plasmid curing. DNA fragments containing the *lacI*-P_trc_-inducible system, *PmCDA1* AID, *ugi*, and the Leu-Val-Ala tag were amplified from the plasmid pSY constructed in our previous study (12), and the gene *SpRY* was amplified from the plasmid pCMV-T7-SpRY-P2A-EGFP (18). The aforementioned modules were assembled to obtain the plasmid pAM4787-SpRY-AID, and then, the working plasmid pBeSpRYc was constructed by mutating SpRY with D10A and H840A. The gRNA cassette targeting specific genome locus was constructed by inverse PCR on the plasmid pTemplate as illustrated in our previous study (14) and was embedded in pBeSpRY *via* In-Fusion assembly, giving the base editing plasmid. To build the *tetR*-P_tet_-inducible system–driven base editor, the regulatory module was amplified from pTet and implemented to pBeSpRY-glnA by replacing the *lacI*-P_trc_ module, generating plasmid pBeSpRY-glnA-Tet. To construct the base editor pBeSpRYc-nblA for *S*. *elongatus* PCC7942, we replaced the dCas9–AID module with dSpRY–AID in pSY plasmid and integrated the gRNA cassette targeting *nblA* to generate the working plasmid.

All plasmids used in this study are listed in Table S4. All gRNA sequences designed are shown in Table S1, and all primers used in this study (ordered from Beijing Genomics Institute) are listed in Table S5. DNA fragments were amplified using PrimeSTAR Max DNA polymerase (Takara Bio Tech) and assembled into the vector using In-Fusion Snap Assembly Premix Kit (Takara Bio Tech). All plasmids were extracted using TIANprep Mini Plasmid Kit (TIANGEN Biotech), verified by Sanger sequencing and quantified using NanoDrop One Microvolume UV–Vis Spectrophotometer (Thermo Fisher Scientific).

### Transformation of *E*. *coli* and *S*. *elongatus*

*E*. *coli*-competent cells were prepared as described in our previous study (36). In brief, freshly cultured *E*. *coli* cells were gently washed with precooled CaCl_2_ (0.1 M), and the cells were resuspended in 0.1 M of CaCl_2_ with 15% (v/v) of glycerol. The competent cells were stored at −80 °C before use. The base-editing working plasmid (60–80 ng) was added into 100 μl MG1655 or Nissle 1917-competent cells, while the same volume of ddH_2_O was added for control group. The mixture was placed on ice for 20 min, heated shock at 42 °C for 1 min, and transferred to 3 ml fresh LB media with 150 μg/ml ampicillin. After cultivation at 30 °C for 1 h, cells were induced for 3 h with different concentrations of IPTG (0, 0.01, 0.05, and 0.1 mM) or aTc (0, 50, and 100 ng/ml) and plated on LB agar plates with ampicillin to obtain transformants.

To transform the cyanobacterium, *S*. *elongatus* PCC7942 cells were cultivated to an absorbance of 0.5 to 0.7 at 730 nm, and then, 15 ml of culture was centrifuged at 5000 rpm for 15 min at 24 °C. After washing with fresh BG-11, the cells were resuspended in 300 μl BG-11, and 2 μg plasmid DNA was added and incubated overnight at 30 °C without light. The samples were then transferred to a culture tube with appropriate antibiotics. IPTG was added to induce base editing after 24 h of cultivation at 30 °C, 120 rpm. After induction culture for 48 h, cell suspensions were plated on BG-11 plates with appropriate antibiotics, and single colonies were randomly selected for analysis.

### Colony PCR and Sanger sequencing

Transformants were randomly selected for colony PCR. The single colony was suspended in 20 μl ddH_2_O, lysed at 100 °C for 10 min, and 1 μl of supernatant was taken as template DNA. The 20-μl PCR system consisted of 10 μl PrimeSTAR Max DNA polymerase, 0.5 μl positive and negative primers, 1 μl template DNA, and 8 μl ddH_2_O. The PCR products were checked by Sanger sequencing. Based on the sequencing results, the editing efficiency was calculated by dividing the number of edited colonies by all colonies that were screened. All editing efficiencies are summarized in Table S6.

### Plasmid curing

After base editing, the edited pure strains were inoculated in LB medium and cultivated overnight at 40 °C. The cells were streaked on LB agar plate for analysis. About six to eight single colonies were randomly selected to check the presence of plasmids, whereas the wildtype stain (without plasmids) and the working plasmid (with plasmid) were employed as controls. To further verify that the plasmid had been eliminated, individual colony was inoculated to LB solid media with or without ampicillin. The plasmid was cured when the strain can grow without antibiotics but cannot grow with antibiotics.

### Phenotypic verification

Both wildtype and the engineered *E*. *coli* strains were cultivated in M9 minimal medium (15.14 g/l Na_2_HPO_4_·12H_2_O, 3.0 g/l KH_2_PO_4_, 0.5 g/l NaCl, 1.0 g/l NH_4_Cl, 0.241 g/l MgSO_4_, 0.011 g/l CaCl_2,_ and 4 g/l glucose solution; solid medium with 1.5% Agar) or in M9 medium supplied with specific l-amino acids to check the growths of the engineered strains. The working concentrations of amino acids added to M9 minimal medium are as follows: 5 mM l-glutamine, 1 mM l-tyrosine, 0.4 g/l l-arginine, and 0.4 g/l l-aspartate. The edited *S*. *elongatus* was evaluated in BG-11 and nitrogen-depleted BG11 media. In nitrogen-limited condition, the wildtype *S*. *elongatus* would exhibit a bleaching phenotype (turning yellow), whereas the edited strain would remain the same green color as in normal BG-11 medium. All edited strains were evaluated after plasmid curing.

### Whole-genome sequencing

Whole-genome sequencing was carried out to assess off-target events in modified *E*. *coli* strains, following previously established method in our laboratory (14). *E*. *coli* cells at the exponential phase were harvested in volumes of 30 to 50 ml. Following the extraction of the genomic DNA of the sample, qualified DNA samples were randomly broken into 350 to 500 bp fragments by Covaris. After library construction, sequencing was performed by Illumina HiSeq instrument. After the process of quality filtering of the original sequencing data, Clean Reads were compared to the reference genome (U00096 in this study) using BWA (Burrow–Wheeler Aligner), and the results were analyzed with QualiMap (qualimap.conesalab.org). The results can be found in the National Center for Biotechnology Information Sequence Read Archive with the accession number PRJNA1122099.

## Data availability

The whole-genome sequencing data are available in the National Center for Biotechnology Information Sequence Read Archive with accession number PRJNA1122099. All other data are available upon reasonable request.

## Supporting information

This article contains supporting information. (11, 18, 23, 24, 32, 33, 34, 37, 38, 39, 40, 41, 42, 43)

## Conflict of interest

The authors declare that they have no conflicts of interest with the contents of this article.
