# Supplementary material for: One-for-all gene inactivation via PAM-independent base editing in bacteria
Source: J Biol Chem. 2024 Dec 18;301(1):108113. doi: 10.1016/j.jbc.2024.108113 (PMC11782819; doi:10.1016/j.jbc.2024.108113)
Supplement: Supporting Information [file mmc1.pdf]

Supporting Information

One-for-all gene inactivation via PAM-independent base editing in bacteria

Xin Li [a], Ying Wei [a], Shu-Yan Wang [a], Shu-Guang Wang [a,b,c], Peng-Fei Xia [a,*]

[a] School of Environmental Science and Engineering, Shandong University, Qingdao 266237, China

[b] Sino-French Research Institute for Ecology and Environment, Shandong University, Qingdao 266237, China

[c] Weihai Research Institute of Industrial Technology, Shandong University, Weihai 264209, China

* Correspondence:

Peng-Fei Xia

School of Environmental Science and Engineering, Shandong University, Qingdao 266237, China. Email: pfxia@sdu.edu.cn

Table S1. gRNA sequences used in this study

gRNA	Target	Strand [a]	PAM	Protospacer
gRNA01	*glnA*	C	NCT	AAGAACAGCACGTCACTATC
gRNA02	*glnA*	C	NTC	ACAGCACGTCACTATCCCTG
gRNA03	*glnA*	C	NCA	CAGCACGTCACTATCCCTGC
gRNA04	*glnA*	N	NTT	ACATACTTTAACTCTCCTGG
gRNA05	*argH*	N	NTT	CCATAACTCTGTTTCCTTAT
gRNA06	*tyrA*	N	NAC	CCATAATAAACCTCTTAAGC
gRNA07	*tyrB*	N	NTT	ACACGCGATGGTTCTCCAGG
gRNA08	*tyrA*	N	NGA	ACATAGATGCCTCGCGCTCC
gRNA09	*aspC*	N	NGT	ACATGACGAGGTTCCATTAT
gRNA10	*nblA*	N	NCA	GCATGGGAGCCTCCGGCACT

[a] C stands for coding strand, and N stands for non-coding strand.

Table S2. Summary of Off-target events in representative reports.

Strains	Effector	Target	Number of off-target events	Sources
E. coli	dCas-CDA	*rpoB*	2	(1)
	dCas-CDA-UL	*rpoB*	30	
Bacillus subtilis	dCas9-AID	*mpr nprB vpr wprA aprE nprE epr bpr*	19	(2)
B. subtilis	CBE4	*sigF*	28	(3)
Pseudomonas putida	CBE-SpRY	-	12 - 30	(4)
Corynebacterium glutamicum	pCoryne-BE3	*ldh*-W134Ter	48	(5)
		actA-W171Ter, *pqo*-Q8Ter, *pta*-W142Ter, *ack*-Q37Ter, *ldh*-W134Ter	184	
C. glutamicum	MACBETH	Cgl0606	9	(6)
Streptomyces coelicolor	CRISPR-cBEST	SCO5087-1	56	(7)
		SCO5092	38	
S. coelicolor	CRISPR-mcBEST	-	27	(8)
		-	27	
		-	92	
S. coelicolor	eSCBE3-NG	PSP_CGG-4	75	(9)
	eSCBE3-NG-HF1	PSP_CGG-4	48	
	eSCBE3-NG-Hypa	PSP_CGG-4	22	
Streptomyces lividans	BE2	*redD*	25	(10)
Agrobacterium tumefaciens	CBE	*recA*[Q26*]	39	(11)
Agrobacterium rhizogenes		*rolB*[R31*]	62	
Clostridium autoethanogenum	multiplex Target-AID	-	15	(12)

Table S3. Strains used in this study

strains	description	sources
E. coli DH5α	Commercial *E. coli* strain for molecular cloning	Takara Bio. Tech.
E. coli MG1655	Wild type strain for the evaluation of the system	Lab stock (CGSC#6300)
E. coli Nissle 1917	Clinical *E. coli* isolate	Ho lab (13)
S. elongatus PCC7942	Model cyanobacterium strain	Lab stock (ATCC33912)
E. coli LX01	MG1655, *glnA* inactivation by dSpRY-AID system with ATG START codon mutating to ATA	This study
E. coli LX02	MG1655, *glnA* inactivation by dCas9-AID system with START codon ATG mutating to ATA	This study
E. coli LX03	MG1655, *argH* inactivation by dSpRY-AID system with START codon ATG mutating to ATA	This study
E. coli LX04	MG1655, *aspC* and *tyrB* inactivated by dSpRY-AID system with START codons ATG and GTG mutating to ATAs	This study
E. coli LX05	MG1655, *tyrA* inactivated by dSpRY-AID system with ATGG mutating to ATAA	This study
E. coli LX06	MG1655, *tyrA* inactivated by dSpRY-AID system with two ATG codons mutating to ATAs	This study
E. coli LX07	MG1655, *tyrB* inactivated by dSpRY-AID system with GTG mutating to ATA	This study
E. coli LX08	Nissle 1917, *argH* inactivation by dSpRY-AID system with START codon ATG mutating to ATA	This study
S. elongatus LX01	PCC7942, *nblA* inactivation by dSpRY-AID system with START codon ATG mutating to ATA	This study

Table S4. Plasmids used in this study

Name	Description	References
pAM4787	*ColE1 ori*, partial sequence from pANS	Lab stock
pSY	*ColE1 ori*, *SmR*, dCas9-PmCDA1-*ugi*	Lab stock
pCMV-T7-SpRY-P2A-EGFP	*ColE1 ori*, *bla*, *SpRY*	Addgene #275392 (14)
pKD46	*oriR101*, *bla*	Lab stock (15)
pTemplate	*pUC ori*, gRNA, *bla*	Lab stock
pTet	*tetR*-P_{tet} *regulation module*	Lab stock
pAM4787-SpRY-AID	*ColE1 ori*, *SmR*, SpRY-PmCDA1-*ugi*	This study
pBeSpRYc	pAM4787*, SmR*, dSpRY-PmCDA1-*ugi*	This study
pBeSpRY	*oriR101*, *bla*, *trc* promoter, dSpRY-PmCDA1-*ugi*	This study
pBeCas9	*oriR101*, *bla*, *trc* promoter, dCas9-PmCDA1-*ugi*	This study
pgRNA01	pTemplate, gRNA-*glnA*-stop-15	This study
pgRNA02	pTemplate, gRNA-*glnA*-stop-19	This study
pgRNA03	pTemplate, gRNA-*glnA*-stop-20	This study
pgRNA04	pTemplate, gRNA-*glnA*	This study
pgRNA05	pTemplate, gRNA-*argH*	This study
pgRNA06	pTemplate, gRNA-*tyrA*	This study
pgRNA07	pTemplate, gRNA-*tyrB*02	This study
pgRNA08	pTemplate, gRNA-*tyrA* 2 loci	This study
pgRNA09	pTemplate, gRNA-*aspC*	This study
pgRNA10	pTemplate, gRNA-*nblA*	This study
pBeSpRY-glnA15	pBeSpRY, gRNA01	This study
pBeSpRY-glnA19	pBeSpRY, gRNA02	This study
pBeSpRY-glnA20	pBeSpRY, gRNA03	This study
pBeCas9-glnA15	pBeCas9, gRNA01	This study
pBeCas9-glnA19	pBeCas9, gRNA02	This study

pBeCas9-glnA20	pBeCas9, gRNA03	This study
pBeSpRY-glnA	pBeSpRY, gRNA04	This study
pBeSpRY-glnA-Tet	*tetR*, P_{tet}-dSpRY-PmCDA1-*ugi*, gRNA04	This study
pBeCas9-glnA	pBeCas9, gRNA04	This study
pBeSpRY-tyrA	pBeSpRY, gRNA06	This study
pBeSpRY-tyrA-2loci	pBeSpRY, gRNA06, gRNA08	This study
pBeSpRY-argH	pBeSpRY, gRNA05	This study
pBeSpRY-aspC	pBeSpRY, gRNA09	This study
pBeSpRY-aspC-tyrB	pBeSpRY, gRNA07, gRNA09	This study
pBeSpRYc-nblA	pBeSpRYc, gRNA10	This study

Table S5. Primers used in this study

Primer	Sequence
**Primers for cloning**	
XIA-LX-247	CAGCTGGGAGGCGACGGAGGTGGAGGAGGTTCTGGAGG
XIA-LX-223	ATGCTGTACTTCTTGTCCATCATGGTCTGTTTCCTGTGTG
XIA-LX-246	CCAGAACCTCCTCCACCTCCGTCGCCTCCCAGCTGAGACA
XIA-LX-225	CACACAGGAAACAGACCATGATGGACAAGAAGTACAGCATCGGCCT
XIA-LX-263	GAAGTACAGCATCGGCCTGGCTATCGGCACCAACTCTGTG
XIA-LX-264	AAGCTCTGAGGCACGATGGCGTCCACATCGTAGTCGGACA
XIA-LX-265	CCGACTACGATGTGGACGCCATCGTGCCTCAGAGCTTTCT
XIA-LX-266	ACAGAGTTGGTGCCGATAGCCAGGCCGATGCTGTACTTCT
XIA-LX-295	ATTCGAAACCGGTATCCGCAGGTGGCACTTTTCGGGGAAA
XIA-LX-298	TGTCTTGCGTCTCTGTCGACGGGTATGGACAGTTTTCCCT
XIA-LX-293	TCCCCGAAAAGTGCCACCTGCGGATACCGGTTTCGAATTG
XIA-LX-297	AGGGAAAACTGTCCATACCCGTCGACAGAGACGCAAGACA
XIA-LX-350	CCATAATAAACCTCTTAAGCGTTTTAGAGCTAGAAATAGC
XIA-LX-351	GCTTAAGAGGTTTATTATGGGCTAGCATTATACCTAGGAC
XIA-LX-352	ACATACTTTAACTCTCCTGGGTTTTAGAGCTAGAAATAGC
XIA-LX-353	CCAGGAGAGTTAAAGTATGTGCTAGCATTATACCTAGGAC
XIA-LX-360	AGCGGATTTGAACGTTGCGAATCCTTGACAGCTAGCTCAG
XIA-LX-361	CCAGCTCGGTCTAGATTGCTCCTTTGAGTGAGCTGATACC
XIA-LX-362	CTGAGCTAGCTGTCAAGGATTCGCAACGTTCAAATCCGCT
XIA-LX-363	CGGTATCAGCTCACTCAAAGGAGCAATCTAGACCGAGCTGG
XIA-LX-396	AAGAACAGCACGTCACTATCGTTTTAGAGCTAGAAATAGC
XIA-LX-397	GATAGTGACGTGCTGTTCTTGCTAGCATTATACCTAGGAC
XIA-LX-398	ACAGCACGTCACTATCCCTGGTTTTAGAGCTAGAAATAGC
XIA-LX-399	CAGGGATAGTGACGTGCTGTGCTAGCATTATACCTAGGAC
XIA-LX-400	CAGCACGTCACTATCCCTGCGTTTTAGAGCTAGAAATAGC
XIA-LX-401	GCAGGGATAGTGACGTGCTGGCTAGCATTATACCTAGGAC
XIA-LX-387	CATCCGGCTCGTATAATGTG
XIA-LX-411	AATGGACAACTCGCTCCGTC
XIA-LX-364	GACGGAGCGAGTTGTCCATT

XIA-LX-389	CACATTATACGAGCCGGATG
XIA-LX-446	CCATAACTCTGTTTCCTTATGTTTTAGAGCTAGAAATAGC
XIA-LX-447	ATAAGGAAACAGAGTTATGGGCTAGCATTATACCTAGGAC
XIA-LX-422	ACATGACGAGGTTCCATTATGTTTTAGAGCTAGAAATAGC
XIA-LX-423	ATAATGGAACCTCGTCATGTGCTAGCATTATACCTAGGAC
XIA-LX-453	ACACGCGATGGTTCTCCAGGGTTTTAGAGCTAGAAATAGC
XIA-LX-454	CCTGGAGAACCATCGCGTGTGCTAGCATTATACCTAGGAC
XIA-LX-404	ACATAGATGCCTCGCGCTCCGTTTTAGAGCTAGAAATAGC
XIA-LX-405	GGAGCGCGAGGCATCTATGTGCTAGCATTATACCTAGGAC
XIA-LX-406	GGTATCAGCTCACTCAAAGGTTGACAGCTAGCTCAGTCCT
XIA-LX-407	AACTGTCCATACCCGTCGACGAGAGCGTTCACCGACAAAC
XIA-LX-408	AGGACTGAGCTAGCTGTCAACCTTTGAGTGAGCTGATACC
XIA-LX-409	GTTTGTCGGTGAACGCTCTCGTCGACGGGTATGGACAGTT
XIA-LX-491	GCATGGGAGCCTCCGGCACTGTTTTAGAGCTAGAAATAGC
XIA-LX-492	AGTGCCGGAGGCTCCCATGCGCTAGCATTATACCTAGGAC
XIA-LX-582	TCAATTCAGGGTGGTTCGACGTCTTAAGACCCAC
XIA-LX-584	GGCCGATGCTGTACTTCTTG
XIA-LX-592	GAAGTACAGCATCGGCCTG
XIA-LX-593	TACTCAGCGTCGGTCATCC
XIA-LX-594	GATGACCGACGCTGAGTAC
XIA-LX-595	ACCACCCTGAATTGACTCTCT
**Primers for verification**	
XIA-LX-258	CTATGAGAAGCTGAAGGGCT
XIA-LX-273	CATCCGGCTCGTATAATGTG
XIA-LX-274	CGGTTCTTGTCGCTTCTGGT
XIA-LX-299	TCGTTCTCATGGCTCACGCA
XIA-LX-300	ATAAGGGCGACACGGAAATG
XIA-LX-364	GACGGAGCGAGTTGTCCATT
XIA-LX-375	CCTACCTACGTAACGGACTA
XIA-LX-391	GACAGCTTATCATCGACTGC
XIA-LX-412	CTCTCATCATACGCAGTGTG
XIA-LX-410	ATGGTTCGTTCTCATGGCTC

XIA-LX-275	AAGCAATCTAGACCGAGCTG
XIA-LX-354	ACTGATGCCAGATCGACCAG
XIA-LX-355	CAGCAATTAACGCTATGCGC
XIA-LX-356	AGGCCAACATAGATGCCTCG
XIA-LX-357	ACCTTCGTATTGGGTGGAGG
XIA-LX-358	GAACGTACCGGATTGTTGGA
XIA-LX-359	CAATCGAGGAGCCGTCAAAC
XIA-LX-384	CCACCTTCATATTGGGTGGA
XIA-LX-385	GAACGTACCGGATTGTTGGA
XIA-LX-386	CAATCGAGGAGCCGTCAAAC
XIA-LX-418	CTTATCCCGGATTCTCAGGA
XIA-LX-448	GGATATCTTCGGCGTCGCTT
XIA-LX-449	CGCGAAAGCAGAACAACTGA
XIA-LX-450	CTCTTCTGCGGTTAACACGC
XIA-LX-419	CAGTTAAGCCCTTCCATCGG
XIA-LX-420	AGGGTCGCGATGAAATACGT
XIA-LX-421	CGGCTTGCAGTTGTGGAATA
XIA-LX-426	GTAGGTTCAGACGACACCGT
XIA-LX-427	TGCGAGCACGTTTGTCATTG
XIA-LX-428	CAGCCTTTTTCACGCTGGTC
XIA-LX-413	CGATGGTGCGCATGATAACG
XIA-LX-493	ACTGCGCATTTTCGTGACAC
XIA-LX-580	ACACTCCGCTATCGCTACGT
XIA-LX-581	AATTGATCTGCTGCCTCGCC
XIA-LX-596	CTCTGTGGTTTATTCACAGC
XIA-LX-259	AATGCTGCTGCCTCTACTTC
XIA-LX-260	GTGTACCAGTTGCCAAGCCA
XIA-LX-204	CGAGATAGCAGTATTGACGG

Table S6. Base editing efficiency in this study

Target	gRNA	Rounds of independent replicates	Number of selected colonies	Editing efficiency %	Colonies with pure edits	Pure colonies after one-round of segregation
**Edited by *lacI-trc*-dSpRY-AID**						
glnA	gRNA01	4	44	59.09 ± 22.88	0	Yes
glnA	gRNA02	4	44	100	12	Yes
glnA	gRNA03	4	44	95.45 ± 9.09	1	Yes
glnA	gRNA04	3	33	90.91	0	Yes
glnA[a]	gRNA04	3	44	90.91 ± 9.09	0	Yes
glnA[b]	gRNA04	3	44	100	0	Yes
glnA[c]	gRNA04	3	44	93.94 ± 10.50	1	Yes
argH	gRNA05	3	33	93.94 ± 5.25	1	Yes
tyrA	gRNA06	3	33	100	20	Yes
tyrA 2 loci	gRNA06, gRNA08	3	33	100	29	Yes
tyrB	gRNA07	3	33	87.88 ± 20.99	2	Yes
aspC-tyrB	gRNA07, gRNA09	3	33	93.94 ± 10.50	1	Yes
**Edited by *tet*-dSpRY-AID**						
glnA[a]	gRNA04	3	44	100	21	Yes
glnA[d]	gRNA04	3	44	96.97 ± 5.25	14	Yes
glnA[e]	gRNA04	3	44	96.97 ± 5.25	18	Yes
**Edited by dCas9-AID**						
glnA	gRNA01	3	33	0	0	Yes
glnA	gRNA02	3	33	0	0	Yes
glnA	gRNA03	3	33	0	0	Yes
glnA	gRNA04	3	33	45.45 ± 9.09	0	Yes

[a] induced concentration with 0 mM IPTG or 0 ng/mL aTc

[b] induced concentration with 0.01 mM IPTG

[c] induced concentration with 0.05 mM IPTG

[d] induced concentration with 50 ng/mL aTc

[e] induced concentration with 100 ng/mL aTc

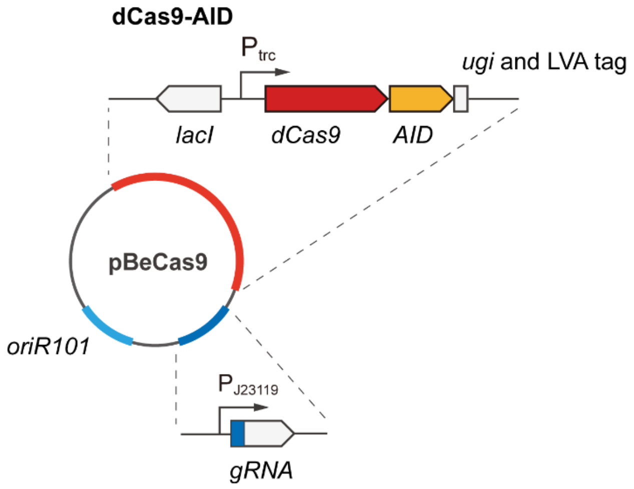

Figure S1. Design of the working plasmids pBeCas9-gRNA. pBeCas9 plasmid contains the dCas9-AID module carrying *dCas9*, *AID*, *ugi* and LVA tag driven by a *lacI*-P_{trc} inducible system. The gRNA cassette is under the control of the constitutive promoter P_{J23119}

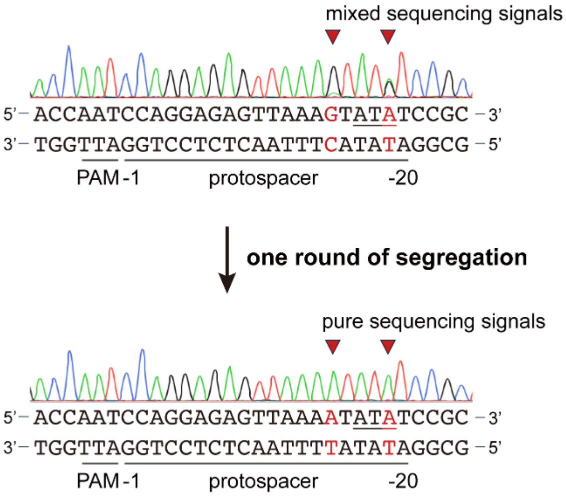

Figure S2. Sequencing results of the edited strain with mixed signals and the sequencing results of the pure edited strain after one more round of segregation. The edited nucleotides are indicated by red arrows and highlighted in red.

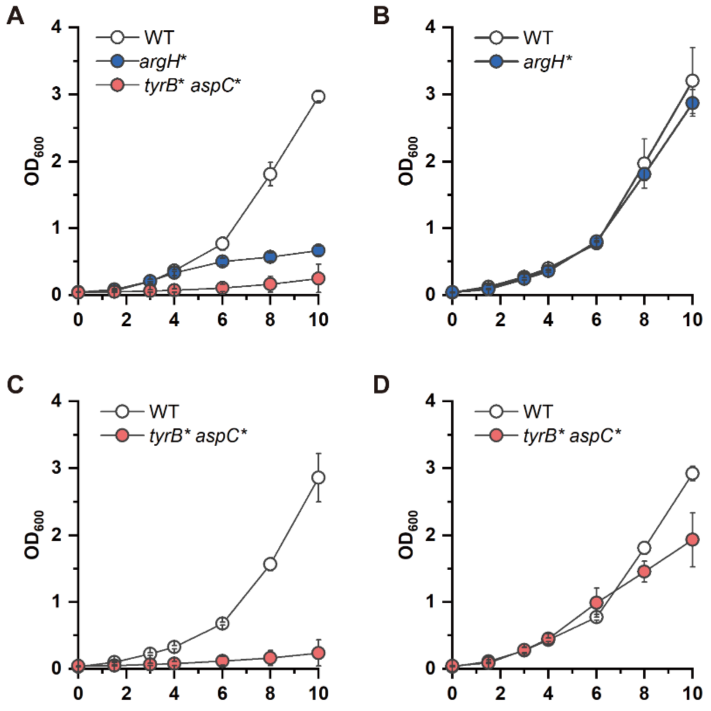

Figure S3. Growth profiles of engineered auxotrophic strains. (A) Growth in M9 medium without extra supplement of amino acids. **(B)** Growth of the WT and *argH* inactivated strains in M9 medium with arginine. Growth of the WT and the *tyrB* and *aspC* double inactivated strains in M9 medium with tyrosine **(C)** and asparate **(D)**. Experiments were performed with three independent replicates and the error bars indicate the standard deviations. All strains were evaluated after plasmid curing.

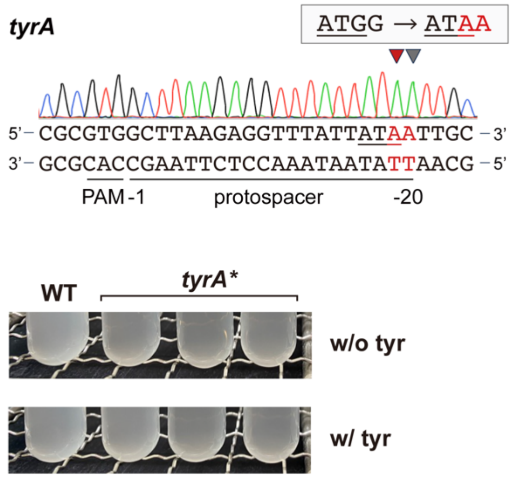

Figure S4. Sequencing result of *tyrA* edited by XSTART, and the phenotypical evaluation of the *tyrA* inactivated strain. The edited nucleotides are indicated by red (intended editing) and grey (bystander editing) arrows and highlighted in red. The wild type strain and three randomly picked clonies carrying designed edits in *tyrA* were cultured in minimal medium with and without 1 mM L-tyrosine.

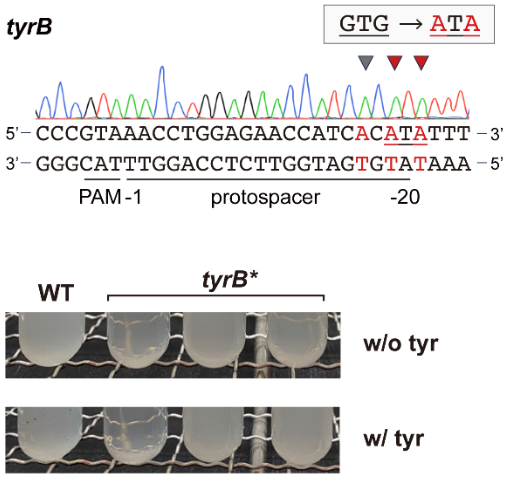

Figure S5. Sequencing analysis of *tyrB* edited by XSTART, and the phenotypical evaluation of the *tyrB* inactivated strain. The edited nucleotides are highlighted in red and the edited loci are indicated by red arrows. The wild type strain and three randomly picked clones carrying desired edits in *tyrB* were cultured in minimal medium with and without 1 mM L-tyrosine.

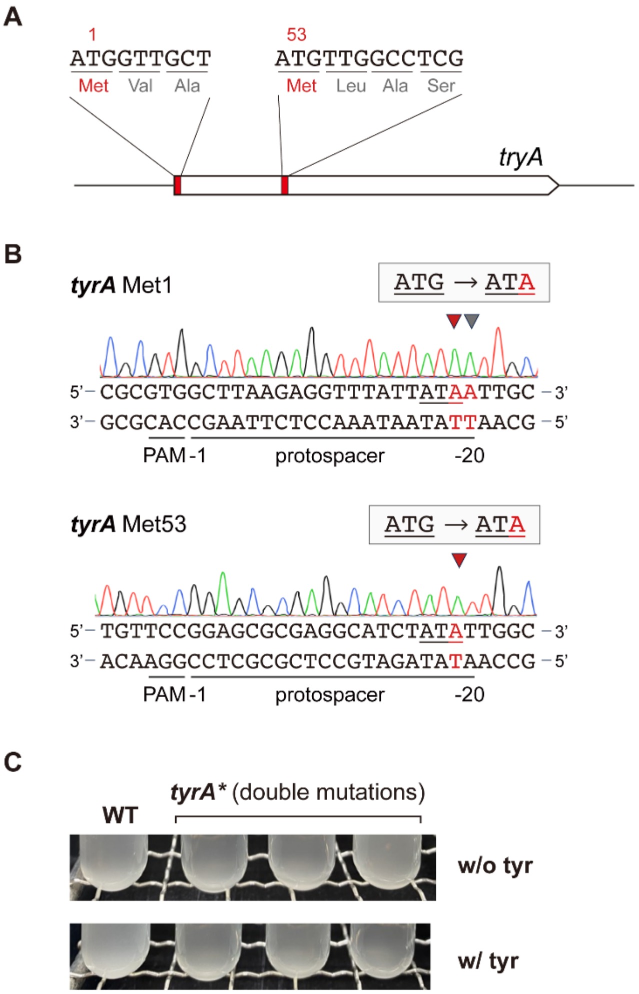

Figure S6. Evaluation of a multiplex XSTART system targeting *tyrA* with tandem gRNAs. **(A)** The two ATG targets coding for Met1 and Met53 in *tyrA*. **(B)** Sequencing of the two edited loci with XSTART. The edited nucleotides are highlighted in red and indicated by red arrow. Grey arrow indicates bystander editing. **(C)** The phenotypical evaluation of the *tyrA* inactivated strain. The wild type strain and three randomly picked clones carrying designed double-edits in *tyrA* were cultured in minimal medium with and without 1 mM L-tyrosine.

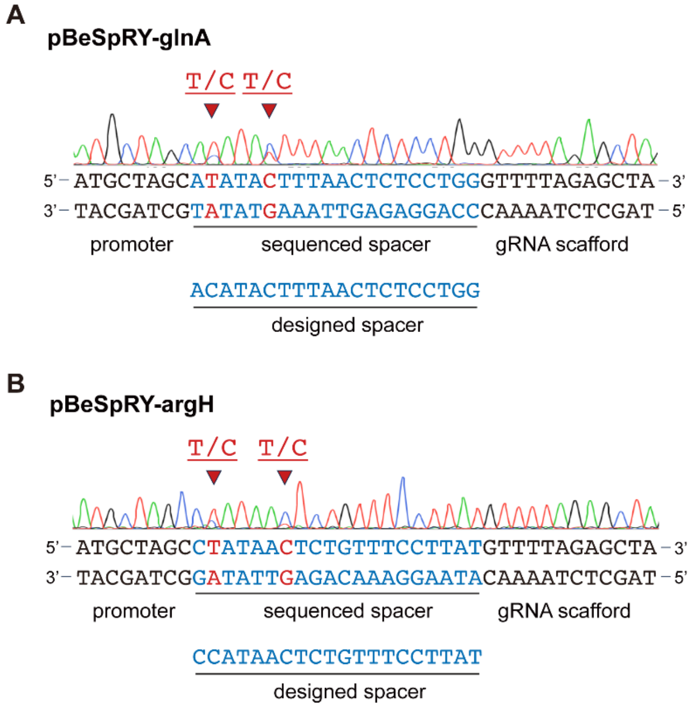

Figure S7. The sequencing results of the gRNA cassette on pBeSpRY-glnA **(A)** and pBeSpRY-argH **(B)**. The designed spacer is highlighted in blue and the mixed sequencing signals of mutated nucleotides are highlighted with red arrows and red font.

References

1. Banno, S., Nishida, K., Arazoe, T., Mitsunobu, H., and Kondo, A. (2018) Deaminase-mediated multiplex genome editing in *Escherichia coli*. *Nat Microbiol.* **3**, 423-429.
2. Yu, S., Price, M. A., Wang, Y., Liu, Y., Guo, Y., Ni, X. *et al.* (2020) CRISPR-dCas9 mediated cytosine deaminase base editing in *Bacillus subtilis*. *ACS Synth Biol.* **9**, 1781-1789.
3. Kim, M. S., Kim, H. R., Jeong, D. E., and Choi, S. K. (2021) Cytosine base editor-mediated multiplex genome editing to accelerate discovery of novel antibiotics in *Bacillus subtilis* and Paenibacillus polymyxa. *Front Microbiol.* **12**, 691839.
4. Kozaeva, E., Nielsen, Z. S., Nieto-Dominguez, M., and Nikel, P. I. (2024) The pAblo.pCasso self-curing vector toolset for unconstrained cytidine and adenine base-editing in Gram-negative bacteria. *Nucleic Acids Res.* **52**, e19.
5. Heo, Y. B., Hwang, G. H., Kang, S. W., Bae, S., and Woo, H. M. (2022) High-fidelity cytosine base editing in a GC-rich *Corynebacterium glutamicum* with reduced DNA Off-target editing effects. *Microbiol Spectr.* **10**, e0376022.
6. Wang, Y., Liu, Y., Liu, J., Guo, Y., Fan, L., Ni, X. *et al.* (2018) MACBETH: Multiplex automated *Corynebacterium glutamicum* base editing method. *Metab Eng.* **47**, 200-210.
7. Tong, Y., Whitford, C. M., Robertsen, H. L., Blin, K., Jorgensen, T. S., Klitgaard, A. K. *et al.* (2019) Highly efficient DSB-free base editing for *Streptomycetes* with CRISPR-BEST. *Proc Natl Acad Sci U S A.* **116**, 20366-20375.
8. Whitford, C. M., Gren, T., Palazzotto, E., Lee, S. Y., Tong, Y., and Weber, T. (2023) Systems analysis of highly multiplexed CRISPR-base editing in *Streptomycetes*. *ACS Synth Biol.* **12**, 2353-2366.
9. Wang, J., Wang, K., Deng, Z., Zhong, Z., Sun, G., Mei, Q. *et al.* (2024) Engineered cytosine base editor enabling broad-scope and high-fidelity gene editing in *Streptomyces*. *Nat Commun.* **15**, 5687.
10. Zhang, Y., Yun, K., Huang, H., Tu, R., Hua, E., and Wang, M. (2021) Antisense RNA interference-enhanced CRISPR/Cas9 base editing method for improving base editing efficiency in

Streptomyces lividans 66. *ACS Synth Biol.* **10**, 1053-1063.

11. Rodrigues, S. D., Karimi, M., Impens, L., Van Lerberge, E., Coussens, G., Aesaert, S. *et al.* (2021) Efficient CRISPR-mediated base editing in *Agrobacterium* spp. *Proc National Acad Sci.* **118**, e2013338118.
12. Seys, F. M., Humphreys, C. M., Tomi-Andrino, C., Li, Q., Millat, T., Yang, S. *et al.* (2023) Base editing enables duplex point mutagenesis in *Clostridium autoethanogenum* at the price of numerous off-target mutations. *Front Bioeng Biotechnol.* **11**, 1211197.
13. Ho, C. L., Tan, H. Q., Chua, K. J., Kang, A., Lim, K. H., Ling, K. L. *et al.* (2018) Engineered commensal microbes for diet-mediated colorectal-cancer chemoprevention. *Nat Biomed Eng.* **2**, 27-37.
14. Walton, R. T., Christie, K. A., Whittaker, M. N., and Kleinstiver, B. P. (2020) Unconstrained genome targeting with near-PAMless engineered CRISPR-Cas9 variants. *Science.* **368**, 290-296.
15. Datsenko, K. A., and Wanner, B. L. (2000) One-step inactivation of chromosomal genes in *Escherichia coli* K-12 using PCR products. *Proc Natl Acad Sci U S A.* **97**, 6640-6645.
