# Supplementary material for: One-for-all gene inactivation via PAM-independent base editing in bacteria
Source: J Biol Chem. 2024 Dec 18;301(1):108113. doi: 10.1016/j.jbc.2024.108113 (PMC11782819; doi:10.1016/j.jbc.2024.108113)
Supplement: Supporting Information [file mmc2.docx]

**Supporting Information Dataset-1**

Summary of off-target evens in strains induced with 0.1 mM IPTG. Overview of the base changes and mutation types and detailed information of off-target editing are included.

**Supporting Information Dataset-2**

Summary of off-target evens in strains induced with 0.01 mM IPTG and 50 ng/mL aTc. Overview of the base changes and mutation types and detailed information of off-target editing are included.
